# Supplementary material for: A Likelihood Approach for Real-Time Calibration of Stochastic Compartmental Epidemic Models
Source: PLoS Comput Biol. 2017 Jan 17;13(1):e1005257. doi: 10.1371/journal.pcbi.1005257 (PMC5240920; doi:10.1371/journal.pcbi.1005257)
Supplement: S1 File — (TAR.GZ) [file pcbi.1005257.s014.tar.gz › HSPH_Online-SI-Revision/MSS10/n1-extreme/n1-extreme_NEW_table8.pdf]

| | | | | | | | | | | | | | | | | | | | | | | | | | | | |
|---|---|---|---|---|---|---|---|---|---|---|---|---|---|---|---|---|---|---|---|---|---|---|---|---|---|---|---|
| 1 | R0 | {2.95019, 14.2854, 0.1} | {2.53075, 11.6597, 0.} | {{2.53075, 11.6597, 0.}, {2.53075, 11.6597, 0.}} | {14.2174, 18.3802, 100.} | inf | 19. | 2.16637 | {1.23685, 3.21995} | 88.598 | 0.974829 | newly | 10. | 0.96 | {0., 2.} | 90.4 | 1.22423 | Reff | 0.253716 | 0.260246 | {0.257456, 0.262584} | 2.57467 | ar | 924. | 898.12 | {896., 900.} | 2.80087 |
| 2 | R0 | {2.67565, 16.946, 0.1} | {2.93978, 19.9817, 0.} | {{2.93978, 19.9817, 0.}, {2.93978, 19.9817, 0.}} | {9.87144, 17.9141, 100.} | inf | 31. | 27.5389 | {25.3256, 29.55} | 11.1649 | 0.0520634 | newly | 14. | 8.77 | {6., 12.} | 37.3571 | 0.22111 | Reff | 0.232782 | 0.265935 | {0.259941, 0.27214} | 14.2425 | ar | 926. | 918.73 | {914., 923.} | 0.819654 |
| 3 | R0 | {2.40066, 16.6543, 0.1} | {2.47127, 17.0304, 0.72} | {{2.45321, 15.7026, 0.}, {2.48143, 19.391, 2.}} | {2.94117, 9.57302, 748.} | inf | 69. | 54.854 | {38.3219, 65.6501} | 20.5014 | 0.10925 | newly | 26. | 20.01 | {12., 27.} | 26.1154 | 0.147552 | Reff | 0.249669 | 0.291826 | {0.266062, 0.329094} | 16.8853 | ar | 916. | 901.89 | {880., 917.} | 1.62118 |
| 4 | R0 | {1.9294, 15.4361, 0.1} | {1.87927, 11.8576, 0.} | {{1.88607, 11.9453, 0.}, {1.88607, 11.9453, 0.}} | {2.5982, 23.1826, 100.} | inf | 64. | 63.941 | {58.6929, 70.1401} | 6.59035 | 0.0296627 | newly | 26. | 32.07 | {26., 38.} | 25.4231 | 0.095371 | Reff | 0.507432 | 0.494217 | {0.482729, 0.504887} | 2.6293 | ar | 796. | 787.3 | {767., 806.} | 1.81156 |
| 5 | R0 | {2.92859, 19.5393, 0.1} | {1.98971, 19.6444, 0.} | {{1.98971, 19.6444, 0.}, {1.98971, 19.6444, 0.}} | {32.0591, 0.537727, 100.} | inf | 46. | 58.5687 | {56.5385, 60.4638} | 27.3232 | 0.104751 | newly | 14. | 18.1 | {13., 23.} | 32.1429 | 0.115909 | Reff | 0.251859 | 0.146107 | {0.14215, 0.149942} | 41.9885 | ar | 927. | 936. | {931., 942.} | 0.973031 |
| 6 | R0 | {1.79622, 12.3148, 0.1} | {1.58389, 10.8293, 3.} | {{1.58389, 10.8293, 3.}, {1.58389, 10.8293, 3.}} | {11.8211, 12.0628, 2900.} | inf | 28. | 50.8178 | {46.0737, 56.4155} | 81.4922 | 0.257308 | newly | 16. | 27.4 | {21., 33.} | 71.25 | 0.227426 | Reff | 0.555032 | 0.45328 | {0.443204, 0.460697} | 18.3326 | ar | 734. | 749.68 | {736., 764.} | 2.24251 |
| 7 | R0 | {2.45855, 17.1776, 1.} | {2.17119, 13.9306, 2.49} | {{2.03831, 13.1693, 2.}, {2.29887, 14.662, 3.}} | {11.688, 18.9027, 149.} | inf | 47. | 34.464 | {17.5345, 51.8309} | 31.7894 | 0.200954 | newly | 18. | 14.44 | {6., 23.} | 35.3333 | 0.21052 | Reff | 0.358948 | 0.342322 | {0.323931, 0.357181} | 4.70273 | ar | 871. | 857.23 | {832., 885.} | 2.43054 |
| 8 | R0 | {2.20009, 15.519, 2.} | {1.73739, 13.0272, 2.} | {{1.73739, 13.0272, 2.}, {1.73739, 13.0272, 2.}} | {21.0309, 16.0563, 0.} | inf | 24. | 1.87879 | {1.09525, 2.79388} | 92.1717 | 1.13247 | newly | 12. | 0.78 | {0., 2.} | 93.5 | 1.42874 | Reff | 0.378415 | 0.337265 | {0.335544, 0.338618} | 10.8745 | ar | 839. | 807. | {805., 810.} | 3.81406 |
| 9 | R0 | {2.06493, 9.6414, 1.} | {1.96576, 10.2488, 1.04} | {{1.96658, 10.2409, 1.}, {1.96658, 10.2409, 1.}} | {4.8026, 6.29975, 4.} | inf | 11. | 23.9294 | {18.8381, 27.6299} | 117.54 | 0.333653 | newly | 7. | 13.12 | {9., 17.} | 87.4286 | 0.259527 | Reff | 0.489388 | 0.440473 | {0.432724, 0.449732} | 9.99524 | ar | 777. | 793.83 | {783., 807.} | 2.1686 |
| 10 | R0 | {2.60775, 19.6936, 1.} | {2.27582, 18.9086, 0.} | {{2.27582, 18.9086, 0.}, {2.27582, 18.9086, 0.}} | {12.7287, 3.98604, 100.} | inf | 57. | 66.4127 | {62.2627, 71.4832} | 16.5135 | 0.0658394 | newly | 14. | 21.64 | {16., 28.} | 54.5714 | 0.180982 | Reff | 0.341615 | 0.27671 | {0.26507, 0.285511} | 18.9994 | ar | 889. | 899.25 | {890., 908.} | 1.18673 |
| 11 | R0 | {1.99168, 8.13555, 1.} | {1.96658, 10.2409, 1.} | {{1.96658, 10.2409, 1.}, {1.96658, 10.2409, 1.}} | {1.26005, 25.878, 0.} | inf | 7. | 18.7994 | {14.5988, 22.5193} | 168.563 | 0.422492 | newly | 6. | 10.88 | {7., 14.} | 81.6667 | 0.245411 | Reff | 0.478003 | 0.448776 | {0.44126, 0.45656} | 6.11453 | ar | 772. | 786.19 | {775., 798.} | 1.88472 |
| 12 | R0 | {1.79304, 9.18941, 0.1} | {1.75352, 7.52476, 3.} | {{1.75445, 7.55154, 3.}, {1.75445, 7.55154, 3.}} | {2.20382, 18.1149, 2900.} | inf | 15. | 6.62661 | {3.43566, 9.71165} | 55.8226 | 0.39339 | newly | 9. | 4.94 | {2., 8.} | 46. | 0.349164 | Reff | 0.505637 | 0.509175 | {0.50352, 0.514391} | 0.880613 | ar | 725. | 715.66 | {708., 725.} | 1.41517 |
| 13 | R0 | {1.84016, 18.3431, 1.} | {1.5102, 16.3861, 3.} | {{1.5102, 16.3861, 3.}, {1.5102, 16.3861, 3.}} | {17.9313, 10.6687, 200.} | inf | 80. | 60.9542 | {54.1413, 67.2866} | 23.8073 | 0.119485 | newly | 33. | 23.64 | {17., 30.} | 28.4848 | 0.154179 | Reff | 0.651417 | 0.563372 | {0.553737, 0.573191} | 13.5158 | ar | 762. | 690.62 | {667., 716.} | 9.36745 |
| 14 | R0 | {1.94221, 9.85971, 1.} | {1.96444, 10.0952, 0.18} | {{1.93539, 9.63533, 0.}, {2.0968, 12.1899, 1.}} | {1.7208, 6.12019, 82.} | inf | 4. | 10.8137 | {5.24298, 25.8461} | 170.892 | 0.359698 | newly | 2. | 6.1 | {3., 12.} | 207. | 0.42387 | Reff | 0.388442 | 0.379055 | {0.372857, 0.384653} | 2.417 | ar | 805. | 812.85 | {803., 836.} | 1.07702 |
| 15 | R0 | {1.78578, 12.0541, 0.1} | {2.2194, 19.1478, 0.} | {{2.2194, 19.1478, 0.}, {2.2194, 19.1478, 0.}} | {24.2819, 58.8486, 100.} | inf | 20. | 87.6254 | {82.2897, 92.8378} | 338.127 | 0.641132 | newly | 12. | 28.93 | {22., 36.} | 141.083 | 0.376135 | Reff | 0.494661 | 0.464686 | {0.452703, 0.476346} | 6.05967 | ar | 732. | 847.94 | {831., 866.} | 15.8388 |
| 16 | R0 | {1.94463, 15.6503, 2.} | {1.70777, 8.49961, 3.} | {{1.70777, 8.49961, 3.}, {1.70777, 8.49961, 3.}} | {12.18, 45.6904, 50.} | inf | 49. | 13.7535 | {9.31259, 17.8991} | 71.9316 | 0.565482 | newly | 20. | 9.42 | {5., 13.} | 52.9 | 0.354355 | Reff | 0.536718 | 0.531539 | {0.524382, 0.539068} | 1.17121 | ar | 757. | 703.65 | {689., 717.} | 7.04756 |
| 17 | R0 | {2.08901, 16.4314, 0.1} | {2.0818, 17.5151, 1.} | {{2.0818, 17.5151, 1.}, {2.0818, 17.5151, 1.}} | {0.345046, 6.59503, 900.} | inf | 74. | 89.9369 | {84.0865, 95.7989} | 21.5364 | 0.0841912 | newly | 26. | 32.09 | {25., 38.} | 24.8077 | 0.092262 | Reff | 0.48465 | 0.449801 | {0.436986, 0.461616} | 7.19071 | ar | 826. | 840.9 | {827., 856.} | 1.90073 |
| 18 | R0 | {2.76419, 18.56, 2.} | {2.86501, 18.4249, 1.} | {{2.86501, 18.4249, 1.}, {2.86501, 18.4249, 1.}} | {3.64725, 0.727677, 50.} | inf | 52. | 60.388 | {58.1588, 62.7372} | 16.1307 | 0.0647125 | newly | 13. | 19.64 | {16., 23.} | 51.2308 | 0.174704 | Reff | 0.243249 | 0.228089 | {0.221322, 0.234408} | 6.23722 | ar | 920. | 935.98 | {930., 942.} | 1.73696 |
| 19 | R0 | {2.3821, 17.1399, 2.} | {2.02156, 12.6705, 3.07} | {{1.99822, 12.2033, 3.}, {1.99822, 12.2033, 3.}} | {15.1511, 26.576, 53.5} | inf | 44. | 14.9974 | {6.89413, 14.4763} | 75.9282 | 0.625169 | newly | 11. | 6.46 | {2., 9.} | 64.9091 | 0.419863 | Reff | 0.32873 | 0.336335 | {0.332715, 0.349604} | 6.29405 | ar | 883. | 839.15 | {827., 847.} | 5.42356 |
| 20 | R0 | {2.78314, 19.6127, 0.1} | {2.2277, 15.0861, 0.} | {{2.2277, 15.0861, 0.}, {2.2277, 15.0861, 0.}} | {19.9572, 23.08, 100.} | inf | 20. | 4.38338 | {3.18033, 5.50248} | 78.0831 | 0.669163 | newly | 5. | 1.71 | {0., 3.} | 65.8 | 0.631427 | Reff | 0.18647 | 0.184045 | {0.181521, 0.186711} | 1.42566 | ar | 943. | 918.52 | {916., 921.} | 2.59597 |
| 21 | R0 | {1.85124, 14.6794, 0.1} | {2.036, 18.765, 2.} | {{2.036, 18.765, 2.}, {2.036, 18.765, 2.}} | {9.98042, 27.8324, 1900.} | inf | 67. | 89.8284 | {84.489, 95.7295} | 34.0723 | 0.126836 | newly | 31. | 30.42 | {24., 37.} | 12.9677 | 0.0584403 | Reff | 0.596099 | 0.609114 | {0.596914, 0.619447} | 2.29328 | ar | 730. | 792.26 | {766., 813.} | 8.52877 |
| 22 | R0 | {2.1028, 14.9852, 2.} | {1.86094, 12.6465, 3.} | {{1.86094, 12.6465, 3.}, {1.86094, 12.6465, 3.}} | {11.5018, 15.607, 50.} | inf | 31. | 10.3702 | {7.71432, 12.625} | 66.5476 | 0.482566 | newly | 11. | 4.68 | {3., 7.} | 57.4545 | 0.404726 | Reff | 0.395326 | 0.388248 | {0.383128, 0.392696} | 1.79061 | ar | 840. | 798.42 | {791., 805.} | 4.95 |
| 23 | R0 | {1.86601, 10.2157, 1.} | {1.94282, 12.1446, 2.} | {{1.94282, 12.1446, 2.}, {1.94282, 12.1446, 2.}} | {4.11604, 18.8822, 100.} | inf | 19. | 57.1569 | {51.311, 62.1391} | 200.826 | 0.477031 | newly | 9. | 28.57 | {23., 34.} | 217.444 | 0.496338 | Reff | 0.546741 | 0.495113 | {0.484552, 0.506127} | 9.44282 | ar | 747. | 790.05 | {771., 809.} | 5.76305 |
| 24 | R0 | {2.34032, 10.0992, 0.1} | {2.52588, 12.2207, 0.} | {{2.52588, 12.2207, 0.}, {2.52588, 12.2207, 0.}} | {7.92904, 21.0069, 100.} | inf | 19. | 19.9734 | {17.0304, 22.3536} | 9.73987 | 0.0406803 | newly | 7. | 9.38 | {6., 13.} | 42. | 0.14813 | Reff | 0.278498 | 0.298122 | {0.29109, 0.304566} | 7.04622 | ar | 887. | 889.56 | {883., 896.} | 0.466742 |
| 25 | R0 | {1.91548, 15.3401, 1.} | {1.67732, 9.44073, 0.} | {{1.67732, 9.44073, 0.}, {1.67732, 9.44073, 0.}} | {12.4335, 38.4572, 100.} | inf | 36. | 23.1265 | {17.4598, 28.5821} | 35.7599 | 0.199307 | newly | 14. | 14.47 | {10., 20.} | 22.0714 | 0.0965107 | Reff | 0.599545 | 0.546594 | {0.537434, 0.556088} | 8.83192 | ar | 765. | 699.79 | {683., 718.} | 8.52418 |
| 26 | R0 | {2.75283, 10.6721, 0.1} | {2.55707, 11.9179, 0.} | {{2.53075, 11.6597, 0.}, {2.53075, 11.6597, 0.}} | {8.05551, 11.6735, 100.} | inf | 11. | 8.65099 | {6.0191, 9.34035} | 43.4006 | 0.203369 | newly | 5. | 4.01 | {2., 6.} | 37.8 | 0.221834 | Reff | 0.225732 | 0.215093 | {0.211629, 0.220113} | 4.71325 | ar | 926. | 918.07 | {914., 920.} | 1.12419 |
| 27 | R0 | {2.08367, 19.7849, 1.} | {1.82505, 2.67773, 0.7} | {{1.70301, 2.24898, 0.}, {1.89552, 3.08261, 1.}} | {12.412, 86.4658, 38.} | inf | 70. | 7.30789 | {2.17554, 13.0024} | 89.5602 | 1.07013 | newly | 16. | 10.26 | {2., 19.} | 46. | 0.342168 | Reff | 0.43132 | 0.492148 | {0.455767, 0.517945} | 14.1029 | ar | 833. | 736.77 | {726., 751.} | 11.5522 |
| 28 | R0 | {2.64334, 17.1765, 0.1} | {2.86095, 19.9929, 2.} | {{2.86095, 19.9929, 2.}, {2.86095, 19.9929, 2.}} | {8.23254, 16.397, 1900.} | inf | 50. | 63.2967 | {60.2981, 66.0164} | 26.5934 | 0.102114 | newly | 17. | 19.54 | {13., 24.} | 21.8824 | 0.0869224 | Reff | 0.227327 | 0.208001 | {0.200213, 0.216305} | 8.51142 | ar | 934. | 941.73 | {936., 948.} | 0.834047 |
| 29 | R0 | {2.87008, 13.3872, 0.1} | {2.66225, 19.9254, 0.} | {{2.66225, 19.9254, 0.}, {2.66225, 19.9254, 0.}} | {7.24125, 48.8392, 100.} | inf | 16. | 5.01417 | {3.70061, 6.26989} | 68.6615 | 0.514077 | newly | 8. | 1.44 | {0., 3.} | 82. | 0.941547 | Reff | 0.172205 | 0.188982 | {0.1855, 0.192442} | 9.74262 | ar | 941. | 930.16 | {928., 932.} | 1.15197 |
| 30 | R0 | {2.06105, 19.6275, 1.} | {1.63746, 10.1332, 0.} | {{1.63746, 10.1332, 0.}, {1.63746, 10.1332, 0.}} | {20.5521, 48.3726, 100.} | inf | 18. | 30.0281 | {25.7539, 33.9666} | 66.8227 | 0.219879 | newly | 6. | 16.79 | {13., 21.} | 179.833 | 0.436958 | Reff | 0.548239 | 0.415869 | {0.408616, 0.422582} | 24.1446 | ar | 747. | 765.91 | {755., 777.} | 2.53681 |
| 31 | R0 | {2.30764, 13.435, 2.} | {2.52926, 15.0181, 1.04} | {{2.47514, 14.5291, 1.}, {2.60465, 15.5928, 1.}} | {9.60383, 11.7836, 48.} | inf | 14. | 26.2466 | {14.2224, 39.2594} | 88.9388 | 0.235661 | newly | 4. | 10.33 | {5., 17.} | 159.25 | 0.373143 | Reff | 0.313839 | 0.31231 | {0.287302, 0.336065} | 6.72555 | ar | 877. | 886.64 | {869., 906.} | 1.62828 |
| 32 | R0 | {2.30565, 11.9237, 0.1} | {2.17882, 11.7575, 1.} | {{2.17882, 11.7575, 1.}, {2.17882, 11.7575, 1.}} | {5.50104, 1.39362, 900.} | inf | 9. | 12.4381 | {10.0536, 14.6215} | 38.3062 | 0.136676 | newly | 6. | 6.19 | {4., 9.} | 30.1667 | 0.140524 | Reff | 0.341236 | 0.314974 | {0.309649, 0.320074} | 7.69628 | ar | 853. | 861.48 | {856., 867.} | 0.994138 |
| 33 | R0 | {2.24031, 18.582, 1.} | {2.2194, 19.1478, 0.} | {{2.2194, 19.1478, 0.}, {2.2194, 19.1478, 0.}} | {0.933321, 3.04471, 100.} | inf | 83. | 85.4485 | {81.0072, 89.9506} | 4.201 | 0.0177865 | newly | 29. | 27.25 | {20., 34.} | 15. | 0.0697228 | Reff | 0.396535 | 0.3874 | {0.376857, 0.397015} | 2.51545 | ar | 864. | 868.5 | {856., 879.} | 0.953704 |
| 34 | R0 | {2.33306, 15.9759, 0.1} | {2.35937, 16.9637, 2.02} | {{2.35866, 16.9062, 2.}, {2.35866, 16.9062, 2.}} | {1.12758, 6.18329, 1920.} | inf | 34. | 61.3088 | {56.7191, 65.5767} | 80.3201 | 0.255426 | newly | 12. | 21.8 | {17., 27.} | 81.6667 | 0.252837 | Reff | 0.354625 | 0.294191 | {0.284038, 0.304809} | 17.0416 | ar | 854. | 897.42 | {888., 907.} | 5.08431 |
| 35 | R0 | {2.48357, 18.3573, 1.} | {2.08003, 13.1443, 2.} | {{2.08003, 13.1443, 2.}, {2.08003, 13.1443, 2.}} | {16.2482, 28.3973, 100.} | inf | 55. | 14.6849 | {11.4049, 17.9086} | 73.3002 | 0.581861 | newly | 20. | 6.62 | {3., 10.} | 66.9 | 0.517926 | Reff | 0.442075 | 0.454103 | {0.447241, 0.460817} | 2.72979 | ar | 835. | 792.96 | {783., 802.} | 5.03473 |
| 36 | R0 | {2.59548, 19.9046, 0.1} | {2.30108, 15.555, 0.} | {{2.30108, 15.555, 0.}, {2.30108, 15.555, 0.}} | {11.3427, 21.852, 100.} | inf | 36. | 31.9255 | {28.3691, 35.0001} | 11.5442 | 0.0544392 | newly | 9. | 11.94 | {8., 16.} | 37.1111 | 0.131057 | Reff | 0.399704 | 0.363743 | {0.356653, 0.37174} | 8.99701 | ar | 863. | 858.76 | {850., 869.} | 0.825029 |
| 37 | R0 | {2.29641, 16.9868, 0.1} | {1.75668, 13.8428, 0.} | {{1.75668, 13.8428, 0.}, {1.75668, 13.8428, 0.}} | {23.5032, 18.5084, 100.} | inf | 41. | 17.2761 | {13.9093, 19.8971} | 57.8631 | 0.379233 | newly | 12. | 7.48 | {4., 11.} | 37.8333 | 0.235313 | Reff | 0.314608 | 0.28234 | {0.277725, 0.288197} | 10.2565 | ar | 877. | 846.77 | {840., 853.} | 3.44698 |
| 38 | R0 | {2.65743, 15.4569, 1.} | {2.56757, 17.005, 0.} | {{2.56757, 17.005, 0.}, {2.56757, 17.005, 0.}} | {3.38133, 10.0154, 100.} | inf | 23. | 52.6295 | {49.7549, 55.487} | 128.824 | 0.359102 | newly | 9. | 18.44 | {13., 24.} | 104.889 | 0.301931 | Reff | 0.318892 | 0.232033 | {0.224527, 0.238899} | 27.2376 | ar | 897. | 924.32 | {917., 931.} | 3.04571 |
| 39 | R0 | {2.67953, 14.5132, 2.} | {2.63798, 15.5858, 0.} | {{2.46794, 14.1192, 0.}, {2.96797, 18.4042, 0.}} | {8.87317, 10.9733, 100.} | inf | 24. | 45.9135 | {29.7043, 73.7532} | 91.3062 | 0.249858 | newly | 6. | 16.78 | {10., 24.} | 180. | 0.421021 | Reff | 0.310825 | 0.243991 | {0.196527, 0.272244} | 21.5023 | ar | 894. | 918.45 | {900., 950.} | 2.74161 |
| 40 | R0 | {1.94594, 11.928, 1.} | {2.14288, 16.6365, 3.} | {{2.14288, 16.6365, 3.}, {2.14288, 16.6365, 3.}} | {10.1206, 39.474, 200.} | inf | 36. | 76.3655 | {71.9915, 80.7291} | 112.126 | 0.326164 | newly | 15. | 28.04 | {22., 34.} | 86.9333 | 0.266396 | Reff | 0.447566 | 0.406364 | {0.396775, 0.415595} | 9.20582 | ar | 796. | 851.97 | {840., 864.} | 7.03141 |
| 41 | R0 | {1.66297, 10.0047, 0.1} | {1.64295, 11.759, 1.} | {{1.64295, 11.759, 1.}, {1.64295, 11.759, 1.}} | {1.2037, 17.5344, 900.} | inf | 25. | 63.3732 | {57.7317, 69.5044} | 153.493 | 0.402757 | newly | 8. | 31.5 | {26., 37.} | 293.75 | 0.590307 | Reff | 0.53215 | 0.4627 | {0.452291, 0.471897} | 13.051 | ar | 716. | 765.38 | {744., 785.} | 6.89665 |
| 42 | R0 | {2.91568, 16.4453, 1.} | {2.96647, 18.4927, 1.8} | {{2.93371, 17.1903, 1.}, {2.99088, 18.9406, 2.}} | {2.43773, 12.8801, 80.} | inf | 25. | 43.2996 | {18.4448, 53.5356} | 84.1975 | 0.261763 | newly | 14. | 13.8 | {6., 19.} | 28. | 0.140864 | Reff | 0.23617 | 0.185529 | {0.156631, 0.256708} | 25.2109 | ar | 919. | 944.94 | {918., 958.} | 2.92057 |
| 43 | R0 | {2.55898, 15.8889, 0.1} | {2.48052, 17.2058, 1.6} | {{2.35866, 16.9062, 0.}, {2.96797, 18.4042, 2.}} | {9.45897, 8.28832, 1540.} | inf | 46. | 47.3032 | {34.6151, 85.9384} | 32.2051 | 0.125889 | newly | 20. | 16.38 | {9., 27.} | 35.1 | 0.179776 | Reff | 0.30196 | 0.284718 | {0.231577, 0.304762} | 6.29045 | ar | 904. | 898.47 | {880., 942.} | 2.31748 |
| 44 | R0 | {2.28649, 15.9017, 0.1} | {2.48365, 17.7848, 0.96} | {{2.49092, 17.9391, 1.}, {2.49092, 17.9391, 1.}} | {8.62265, 12.7577, 868.} | inf | 33. | 39.241 | {35.9262, 42.3299} | 18.9121 | 0.0743958 | newly | 11. | 13.67 | {9., 18.} | 30.2727 | 0.112019 | Reff | 0.324682 | 0.337181 | {0.329458, 0.346126} | 4.1573 | ar | 880. | 881.8 | {872., 893.} | 0.715909 |
| 45 | R0 | {2.65121, 18.6339, 0.1} | {2.62812, 15.6423, 1.01} | {{2.60082, 15.3681, 1.}, {2.60786, 15.5255, 1.}} | {2.54043, 16.2005, 910.} | inf | 63. | 40.0685 | {34.5158, 45.0658} | 36.3992 | 0.199958 | newly | 16. | 15.12 | {10., 21.} | 21. | 0.0984118 | Reff | 0.230655 | 0.288616 | {0.276473, 0.300212} | 25.1286 | ar | 928. | 904.7 | {894., 915.} | 2.51078 |
| 46 | R0 | {2.52291, 18.0251, 2.} | {2.30278, 18.9949, 1.96} | {{2.29738, 19.0355, 2.}, {2.29738, 19.0355, 2.}} | {8.79586, 5.60679, 2.} | inf | 48. | 31.2977 | {27.0392, 33.6393} | 38.1879 | 0.205013 | newly | 15. | 9.98 | {7., 13.} | 37.8667 | 0.2142 | Reff | 0.32041 | 0.330605 | {0.324755, 0.339512} | 4.47107 | ar | 894. | 870.49 | {860., 879.} | 2.82215 |
| 47 | R0 | {2.22618, 17.1225, 0.1} | {2.1968, 19.9579, 3.} | {{2.1968, 19.9579, 3.}, {2.1968, 19.9579, 3.}} | {1.3198, 16.5596, 2900.} | inf | 64. | 40.6795 | {36.6322, 44.8396} | 36.4382 | 0.198015 | newly | 23. | 12.8 | {9., 16.} | 44.3478 | 0.266812 | Reff | 0.454141 | 0.499377 | {0.489915, 0.508137} | 9.96092 | ar | 850. | 805.54 | {790., 821.} | 5.23059 |
| 48 | R0 | {2.04405, 16.073, 1.} | {1.72327, 13.2214, 4.} | {{1.69755, 12.7129, 4.}, {1.77361, 14.2286, 4.}} | {15.6933, 17.7417, 300.} | inf | 45. | 38.595 | {24.1713, 61.226} | 35.2006 | 0.178231 | newly | 18. | 17.33 | {9., 27.} | 32.6111 | 0.153098 | Reff | 0.533497 | 0.460266 | {0.43403, 0.478136} | 13.7267 | ar | 779. | 761.04 | {731., 799.} | 3.59949 |
| 49 | R0 | {2.84396, 18.097, 1.} | {2.95026, 17.6413, 2.} | {{2.8833, 17.1847, 2.}, {2.96398, 17.7348, 2.}} | {3.73789, 2.51814, 100.} | inf | 41. | 32.1331 | {23.8805, 36.4153} | 21.6266 | 0.109823 | newly | 16. | 10.8 | {7., 14.} | 32.875 | 0.185429 | Reff | 0.170638 | 0.203066 | {0.191311, 0.219957} | 19.0042 | ar | 952. | 939.34 | {932., 947.} | 1.33613 |
| 50 | R0 | {1.85341, 18.0975, 4.} | {1.6568, 10.5289, 5.} | {{1.6568, 10.5289, 5.}, {1.6568, 10.5289, 5.}} | {10.6082, 41.8212, 25.} | inf | 40. | 19.5717 | {14.2286, 24.1154} | 51.0708 | 0.318405 | newly | 17. | 10.67 | {7., 14.} | 37.4706 | 0.221552 | Reff | 0.498567 | 0.479524 | {0.471898, 0.488066} | 3.81962 | ar | 754. | 727.87 | {713., 743.} | 3.49735 |
